# Bloodborne HIV infections in sub-Saharan Africa: evidence of clusters and response options

**DOI:** 10.3389/fpubh.2026.1779450

**Published:** 2026-02-18

**Authors:** David Gisselquist

**Affiliations:** Independent Researcher, Hershey, PA, United States

**Keywords:** Africa, bloodborne, epidemiology, HIV, nosocomial, prevention

## Abstract

Recent papers report HIV sequence clusters in sub-Saharan Africa best explained by bloodborne transmission. Based on these clusters and on persistent reports of HIV infections best explained by bloodborne transmission, this brief considers three policy challenges for regional governments: (a) whether to warn the public about HIV risks during health care; (b) how to find and stop HIV transmission through medical procedures; and (c) whether to punish careless healthcare workers. Consideration of these challenges leads to three recommendations: Governments can warn the public about risk during health care are by inviting patients attending medical facilities suspected to have caused one or more recognized infections to come for tests. Governments that do so can find and stop bloodborne risks. Not punishing healthcare staff facilitates such investigations.

## Introduction

During August–September 1985, in one of the first studies in the region to use HIV tests, 16 (6.2%) of 258 inpatient children aged 2–24 months in Mama Yemo hospital in Kinshasa, Zaire (currently the Democratic Republic of the Congo), were HIV-positive with HIV-negative mothers; the study team considered medical injections to be the most important risk factor (1). Subsequently, surveys and studies have persistently reported HIV infections in sub-Saharan Africa best explained by bloodborne transmission. For example, in a 2015 national survey in Mozambique, at least 33% of HIV-positive children aged 6–23 months had an HIV-negative mother (2). Similarly, surveys and studies reported teens and adults with infections not explained by self-reported sexual behavior. For example, a study in South Africa that followed women aged 13–26 years during 2011–17 observed 44 incident infections in women who claimed never having had vaginal or anal sex (3).

Evidence from several recent studies suggests that bloodborne HIV transmission in sub-Saharan Africa may at times create outbreaks of linked infections. Studies that sequence HIV in blood samples from multiple people and then look for similarities among sequences can say (with varying levels of confidence) which infections are linked by direct or indirect transmission within some specified period. A recent review found 16 such studies that sequenced HIV in blood collected from at least 100 adults during random or comprehensive general population surveys in communities in sub-Sahara Africa (4). Three of 16 studies (5–7) identified a total of 14 HIV sequence clusters from too many infections (8–75 infections each) that were too closely linked by transmission (estimated within *circa* 3–7.5 years) to be explained by heterosexual sex. Awareness of these 14 clusters guides assessment and recommendation of policies to find and stop HIV bloodborne transmission during skin-piercing medical and cosmetic procedures in sub-Saharan Africa.

### HIV sequence clusters in sub-Saharan Africa

A study in South Africa sequenced HIV in blood collected during 2010–2014 from a 15% random sample of adults (aged ≥15 years) in a study community (5). The study found 75 adults with similar sequences, estimating that HIV from one adult in August 2011 reached and infected another 74 over 37 months through September 2014. If everyone infected in this cluster transmitted HIV consistently at the same rate after infection, that rate was once every 6 months (infections doubled 6.2 times in 37 months) or 2.0 per year. Realistically, some transmitted much faster at times. Furthermore, the recognized cluster of 75 infections was likely part of a much larger cluster: infections came from a 15% random sample of adults in the study community; the study did not test children; the observed cluster centered on a town at the edge of the community and likely extended outside; and transmission was ongoing when blood collection ended. The average transmission rate within that larger cluster would have been much faster than 2.0 per year.

A study in Uganda deep sequenced HIV in blood collected during 2011–2014 from adults aged 15–49 years in 40 communities across Rakai District (6). The study found 12 sequence clusters linking 8–19 infections (4 with 8 sequences; 5 with 9; and one each with 10, 12, and 19 sequences). Among 1,334 sequences in the study that clustered with one or more others, 96% had not more than 1.0% genetic distance in *gag+pol* genes with at least one other sequence [Appendix B in (6)] and were therefore linked by transmission within *circa* 5 years (8). As in the South African study, these 12 clusters were likely parts of larger clusters; the study tested only 5% of Rakai District’s population (9). Furthermore, the unreported estimated time from first to last recognized infection in at least some of these clusters was likely less than 5 years. Rough (and likely conservative) rates of transmission within these clusters can be estimated by assuming the study found all infections in each cluster and that the first transmission in each cluster happened 5 years before the last recognized infection. With these assumptions, the numbers of infections in each cluster subsequently doubled 2–3.2 times in 5 years, or approximately once every 1.6–2.5 years, to reach 8–19 linked infections; and average rates of transmission within these 12 clusters ranged from 0.4–0.7 per year.

A study in Cameroon reported HIV sequences from 10 women in four villages along a road with not more than 1.5% genetic distance in *pol* genes (7), suggesting transmission to all women occurred within *circa* 7.5 years (8). If one man was responsible, he would have had to transmit on average to one woman every 9 months, an average rate of 0.75 per year. If more than one man was responsible—if one or more of the 10 women infected men who subsequently infected other women—average transmission could be a bit lower. Likely the cluster was larger, including women not tested, which would entail faster transmission. Furthermore, according to Figure 3 in Edoul et al. (7) the estimated time for transmission to all 10 women was less than 7.5 years.

### Annual rate of HIV transmission between discordant couples in sub-Saharan Africa

The best information on the annual rate of heterosexual transmission in sub-Saharan Africa between persons with frequent sexual contact comes from six studies (Table 1) that followed discordant married or cohabiting couples in which many or most partners did not know their HIV status and few used condoms. In the study in Masaka, Uganda, 1989–97, for example, less than 10% of participants received their test results and none of the HIV-negative partners in discordant couples reported condom use (11). Combining data from these six studies, the average rate of incidence in the initially HIV-negative partner was 0.099 per year (9.9 per 100 person-years) (Table 1).

**Table 1:** HIV incidence in sero-discordant couples in Africa in which most partners did not know their HIV status and/or did not use condoms.

Country, years	Number of incident infections in discordant couples	Total person-years of follow-up	Incidence rate per 100/Person years
Rakai, Uganda, 1989–90 (10)	3	27*	7.3
Masaka, Uganda, 1989–97 (11)	34†	441†	7.7
Rakai, Uganda, 1990–91 (12)	6	66	9.0
Mwanza, Tanzania, 1991–95 (13)	9‡	97‡	9.3
Mwanza, Tanzania, 1991–96 (14)	9§	141§	6.4
Rakai, Uganda, 1994–98 (15)	90¶	760¶	11.8
Totals	151	1,532	9.9

*Calculated from reported incidence rate. †Includes 6 infections in an estimated 85 years of follow-up in spouses of 29 people who seroconverted during follow-up. ‡Includes 3 infections in an estimated 16.5 years of follow-up in spouses of 18 people who seroconverted during follow-up. §Includes data from 5 couples that became discordant during follow-up. ¶Includes data from an undisclosed number of initially HIV-negative couples who became discordant during the first three of four follow-up rounds [two papers (24, 25) discuss incidence in subsets of couples reported in this study].Source: see references in each row.

These studies included circumcised and intact men, people with sexually transmitted infections, people with old and new infections (see notes to Table 1), and no doubt some penile-anal sex. Since some incidence likely came from risks other than sex with the infected spouse, 0.099 per year likely overestimates heterosexual partner-to-partner transmission in these six studies and, more generally, in persons with frequent heterosexual exposure to HIV; it is in any case far less than required to generate any of the 14 sequence clusters discussed above.

### Other risks?

Considering that many nosocomial HIV outbreaks have been reported outside sub-Saharan Africa (16), and that nosocomial outbreaks of other bloodborne viruses have been reported in Africa (17), it would be absurd to assume that none of the 14 reported HIV sequence clusters came from skin-piercing medical (and possibly some cosmetic) procedures. Likely most if not all came from such risks, but that stronger hypothesis is not necessary to guide policies to prevent nosocomial HIV.

Two risks that are known to transmit HIV much faster than heterosexual sex and that can and do generate clusters of HIV infections are injection drug use (IDU) and anal sex among men who have sex with men (MSM). None of the three studies reported IDU or MSM behaviors. According to a recent estimate, IDU accounted for only 1.3% and MSM for 3.4% of incidence among adults aged 15–49 in sub-Saharan Africa in 2022 (18, 19). Interventions proposed below to find and stop non-IDU bloodborne risks would respond to infections suspected from such risks and not from IDU or MSM risks.

## Policy options and implications

### Whether to warn the public about the risk that medical procedures might transmit HIV

The World Medical Association’s (WMA) Declaration of Lisbon on the Rights of the Patient enjoins health professionals to provide patients with (article 9) “health education that will assist him/her in making informed choices about personal health and about the available health services” (20). In countries with persistent reports of unexplained HIV infections, article 9 puts government health staff on the spot: not warning the general public about risks to get HIV during health care denies patients’ rights, while simply warning without at the same time trying to find and stop risks acknowledges that government health staff are not doing what they can to protect patients.

For government health program managers, one solution to this dilemma is to investigate suspected nosocomial infections by inviting others attending suspected transmitting facilities to come for HIV tests. Such invitations warn the public that medical procedures may have transmitted HIV; at the same, by inviting people for tests, governments health staff are doing what is required to find and stop risks. After such warnings and efforts to find and stop risks, patients will have more information to assess their remaining risks.

### How to stop HIV transmission in health facilities

Considering inefficient heterosexual HIV transmission, frequent HIV testing to find people with new infections and putting them on antiretroviral treatment (ART) stops most heterosexual transmission. Similarly, when frequent testing finds someone infected from a high-risk event—non-IDU bloodborne risk, IDU, or anal sex among MSM—putting them on ART will stop most of their transmission through those risks as well. However, people found with infections from high-risk events may be linked to others infected from the same risks. Putting the one found infected on ART does not stop others in the group with unrecognized infections from continuing to transmit through high-risk events. Hence, when one or more people in a community are found with HIV suspected to have come from health care, testing others attending suspected transmitting facilities is necessary to see if they could be part of an ongoing outbreak; if so, treating others found infected and reminding and retraining the facilities’ healthcare providers to practice standard precautions can stop ongoing outbreaks. Tracing and testing could be extended as well to clients at suspected cosmetic facilities.

Scores of governments outside sub-Saharan Africa have responded to one or more people with suspected nosocomial HIV infections by testing others who attended medical facilities suspected to have caused the infection(s). During 1986–2021, 12 investigations of suspected nosocomial HIV infections in 11 countries found outbreaks with from 100 to as many as 100,000 infections (16). For example, in November 1988, the central government of the former Soviet Union received reports of two unexplained HIV infections in Elista, a city in southern Russia (21). Government’s investigation, beginning within the month (22), found that hospitals had spread HIV from a child infected by its mother and hospitalized in May 1988 to 265 children and one adult through August 1989 (23). During the outbreak, infections doubled more than eight times in 16 months, doubling on average every 2 months. If everyone infected in the outbreak transmitted at the same rate to the end of the outbreak, the average rate of transmission would have been once every 2 months, or six times per year. That rate is sufficient to generate the 14 reported sequence clusters recognized in sub-Saharan Africa.

The 12 large nosocomial HIV outbreaks investigated outside sub-Saharan Africa illustrate several reasons nosocomial infections sometimes occur in clusters (16). One reason is repeated treatments (e.g., during antenatal care and delivery, dental care, or in-hospital care): people infected during a medical procedure who return for another weeks or months later with a new infection would have a high blood viral load favoring onward transmission if skin-piercing equipment is reused without sterilization. A second reason is that when staff in one facility are careless about standard precautions, staff in other facilities in the region may be similarly careless, as seen, for example, in orphanages and health facilities throughout Romania in 1986–92 and in hundreds of plasma buying enterprises in China during 1990–94 (16).

Investigating suspected nosocomial HIV infections goes beyond what the Joint United Nations Programme on HIV/AIDS (UNAIDS) and the World Health Organization (WHO) recommend to prevent HIV infections in sub-Saharan Africa. UNAIDS’ recent recommendations for HIV prevention in the region do not mention non-IDU bloodborne transmission, implicitly advising everyone to ignore it (19). WHO on the other hand acknowledges that health care remains a threat to transmit HIV and recommends standard precautions to protect patients (24). Standard precautions are, of course, the answer; but the issue WHO does not address is what could be done differently to overcome decades of failure for those same recommendations to stop nosocomial transmission in some countries and communities.

The most ambitious previous attempt by WHO to address HIV transmission through contaminated medical instruments in sub-Saharan Africa and elsewhere was the Safe Injection Global Network (SIGN), which began in 1999. SIGN responded to model-based estimates that up to 5% of HIV incidence in sub-Saharan Africa was from unsafe medical injections (25). Recognizing that sterilization of medical instruments (standard precautions) was unreliable, WHO and SIGN promoted shifting medical injections to single-use syringes designed to break after use (26). In doing so, WHO and SIGN left an unresolved problem—sterilization for other medical instruments remained unreliable. In effect, WHO recognized that decades of advising, teaching, and recommending standard precautions had failed to ensure that health staff strictly implemented standard precautions.

The investigations proposed in this brief would find and fix deficiencies in standard precautions in specific facilities through pointed advice and retraining. The bigger impact may be from publicity: investigations that invite patients and clients to come for HIV tests and then find and report more infections from medical procedures get into the press, thereby alerting other healthcare staff to be careful to strictly implement standard precautions so as not to infect their patients, and warning the general public to be aware and alert as well.

### Whether to prosecute persons responsible for lapses in standard precautions

The key to preventing nosocomial transmission is to motivate front-line healthcare staff to follow standard precautions. Generating fear—threatening to punish staff found to have infected patients—is one way to motivate. Another way is to trust and engage healthcare providers’ goodwill for patients’ safety by warning providers that what they have been doing may have infected patients and what to do differently. A Charlie Chaplin quote endorses this approach: “You need Power, only when you want to do something harmful otherwise Love is enough to get everything done” (27).

If the focus is on investigating to stop blood-borne transmission, threatening punishment can be counterproductive. For best results in the shortest time, investigators will want healthcare staff to explain their procedures and to identify previous patients. If staff feel threatened, they may not cooperate, which could derail or delay an investigation.

Since blood-borne transmission has likely occurred in thousands of medical settings across sub-Saharan Africa over more than four decades, punishing one or more front-line health care providers for specific infections would be scapegoating, not justice. Furthermore, it would be practically impossible to determine who was responsible for specific lapses in standard precautions resulting in specific infections. Assessing anyone to pay compensation would be similarly unfair and unrealistic; in any case, everyone infected from any risk already has access to free ART.

What has happened outside sub-Saharan Africa provides some perspective. For example, I have seen no reports of any healthcare staff punished after medical procedures infected an estimated 10,000 children throughout Romania during 1986–92 or 100,000 people selling blood plasma in China during 1990–94. On the other hand, governments in Kyrgyzstan, Kazakhstan, and elsewhere punished some healthcare staff after investigations discovered outbreaks with 100–300 infections. In all cases, findings from investigations motivated initiatives that stopped nosocomial transmission (16). Latest reported rates of adult HIV prevalence in Romania and China are 0.1–0.2% and in Kazakhstan and Kyrgyzstan are 0.3% (28, 29).

## Actionable recommendations

Three actionable recommendations follow directly from discussions of the three policy options considered in previous sub-sections.

1. Warn the public about HIV risks during health care by responding to suspected nosocomial infections by inviting people attending suspected healthcare facilities to come for tests and reporting numbers and ages of those found with unexplained infections (but not their names).
2. Investigate infections suspected to have come from skin-piercing healthcare or cosmetic procedures by testing others attending suspected source facilities. In communities with high rates of HIV prevalence, HIV sequencing may be needed to identify at least some of the infections linked through skin-piercing procedures. When testing finds more attendees with infections best explained by skin-piercing procedures, investigators can identify specific procedures and facilities responsible by working backward from who was infected. That information can guide interventions to ensure standard precautions. (In the case of suspected IDU and MSM infections, similar investigations could invite and test contacts. Because these behaviors are often stigmatized or even illegal, care is needed to gain trust and to protect anonymity of people traced and tested.)
3. Do not prosecute healthcare staff and managers for suspected or even acknowledged careless implementation of standard precautions if they cooperate with investigations. This facilitates rapid and thorough investigations to find and treat victims and to correct failures in standard precautions.

## Conclusion

Beginning in 2016, WHO urged governments to help people who test HIV-positive tell their sex partners, thereby endorsing tracing and testing heterosexual partners (30). The proposals in this paper propose similar tracing and testing as a response to infections suspected from skin-piercing procedures in health care or cosmetic facilities.
